# A Reevaluation of the Voluntary Medical Male Circumcision Scale-Up Plan in Zimbabwe

**DOI:** 10.1371/journal.pone.0140818

**Published:** 2015-11-03

**Authors:** Susanne F. Awad, Sema K. Sgaier, Gertrude Ncube, Sinokuthemba Xaba, Owen M. Mugurungi, Mutsa M. Mhangara, Fiona K. Lau, Yousra A. Mohamoud, Laith J. Abu-Raddad

**Affiliations:** 1 Infectious Disease Epidemiology Group, Weill Cornell Medical College in Qatar, Cornell University, Qatar Foundation, Education City, Doha, Qatar; 2 Integrated Delivery, Global Development Program, Bill & Melinda Gates Foundation, Seattle, Washington, United States of America; 3 Department of Global Health, University of Washington, Seattle, Washington, United States of America; 4 AIDS and TB Programme, Ministry of Health and Child Care, Harare, Zimbabwe; 5 Department of Healthcare Policy and Research, Weill Cornell Medical College, Cornell University, New York, New York, United States of America; 6 Vaccine and Infectious Disease Division, Fred Hutchinson Cancer Research Center, Seattle, Washington, United States of America; International AIDS Vaccine Initiative, UNITED STATES

## Abstract

**Background:**

The voluntary medical male circumcision (VMMC) program in Zimbabwe aims to circumcise 80% of males aged 13–29 by 2017. We assessed the impact of actual VMMC scale-up to date and evaluated the impact of potential alterations to the program to enhance program efficiency, through prioritization of subpopulations.

**Methods and Findings:**

We implemented a recently developed analytical approach: the age-structured mathematical (ASM) model and accompanying three-level conceptual framework to assess the impact of VMMC as an intervention. By September 2014, 364,185 males were circumcised, an initiative that is estimated to avert 40,301 HIV infections by 2025. Through age-group prioritization, the number of VMMCs needed to avert one infection (effectiveness) ranged between ten (20–24 age-group) and 53 (45–49 age-group). The cost per infection averted ranged between $811 (20–24 age-group) and $5,518 (45–49 age-group). By 2025, the largest reductions in HIV incidence rate (up to 27%) were achieved by prioritizing 10–14, 15–19, or 20–24 year old. The greatest program efficiency was achieved by prioritizing 15–24, 15–29, or 15–34 year old. Prioritizing males 13–29 year old was programmatically efficient, but slightly inferior to the 15–24, 15–29, or 15–34 age groups. Through geographic prioritization, effectiveness varied from 9–12 VMMCs per infection averted across provinces. Through risk-group prioritization, effectiveness ranged from one (highest sexual risk-group) to 60 (lowest sexual risk-group) VMMCs per infection averted.

**Conclusion:**

The current VMMC program plan in Zimbabwe is targeting an efficient and impactful age bracket (13–29 year old), but program efficiency can be improved by prioritizing a subset of males for demand creation and service availability. The greatest program efficiency can be attained by prioritizing young sexually active males and males whose sexual behavior puts them at higher risk for acquiring HIV.

## Introduction

Zimbabwe is one of 14 countries that are scaling up voluntary medical male circumcision (VMMC) as part of implementation of a comprehensive package of HIV prevention and treatment services including HIV testing and counseling and antiretroviral therapy (ART) among others [1, 2]. With high HIV prevalence (about 15% in 2011 [3]), and a low male circumcision rate (9.1% [3]), Zimbabwe has the potential to avert a higher proportion of new HIV infections than other countries scaling up VMMC [1, 2, 4]. An earlier modeling study predicted that, by reaching 80% VMMC coverage among 15–49 year old males within five years and subsequently maintaining coverage at this level, Zimbabwe can avert 42% of new HIV infections by 2025 (about 600,000 infections) [4].

In light of these results, Zimbabwe adopted VMMC as a key HIV strategy in 2009 and created a steering committee to undertake advocacy, protocol development, and a pilot study of the VMMC program in the country [5, 6]. Unlike the other 13 countries scaling up VMMC, the current VMMC program in Zimbabwe targets 13–29 year old. The program aims to reduce HIV incidence by circumcising 80% of males in this age bracket between 2011 and 2017, for a total of 1.3 million circumcisions [7, 8]. As of the end of 2013, 204,310 adolescent and adult males have been circumcised against the six-year target of 1.3 million—that is, 16% of the national target [8, 9]. Furthermore, less than 80% of completed VMMCs by the end of 2013 were within the targeted age group (13–29 year old), 20% were among adults older than 30 years of age, and 29% were among adolescents from 10–14 years old [9]. Though the VMMC program in Zimbabwe has made substantial progress, several challenges, including availability of adequate resources, are emerging. Increased focus on program efficiency through evaluation and potential modification of the current scale-up plan is needed.

Because the country is developing a costed operational plan for the VMMC program, it is critical to reevaluate the current program scale-up plan, by estimating the epidemic impact of the scale-up to date, and by determining whether making subpopulations a priority for intervention could improve program efficiency. Program efficiency is defined as optimizing program impact (the number of HIV infections averted) and minimizing program cost. Therefore, the objectives of this study are to *1*) estimate the population-level epidemiological impact of the VMMC program to date, and *2*) explore whether Zimbabwe has an opportunity to enhance the program’s efficiency by focusing effort on subpopulations.

## Methods

We implemented a recently developed analytic approach to address these questions and investigate gains in VMMC program efficiency. The approach is expressed in terms of the age-structured mathematical (ASM) model and accompanying three-level conceptual framework [10]. Detailed description of the ASM model and the three-level conceptual framework can be found in Awad et al. [10].

### Mathematical model structure

Briefly, the ASM model is a population-level deterministic compartmental model that consists of a set of coupled nonlinear differential equations [10]. The model stratifies the heterosexual population into compartments according to sex, circumcision status, age group, sexual risk group, HIV status, and stage of infection. The efficacy of VMMC against HIV acquisition among males is modeled as a proportional reduction in the risk of HIV acquisition among circumcised males. HIV progression in the model is divided into the three stages of acute, chronic, and advanced infection. The model stratifies the population into 20 age groups, with each group representing a five-year age band (0–4, 5–9… 95–99). The model incorporates six sexual risk groups to account for heterogeneity in sexual risk behavior in the population [11–14]. The model is fitted to HIV prevalence time series data using a nonlinear least-square fitting method and is implemented in MATLAB [15].

### Three-level conceptual framework

The three-level conceptual framework takes into account epidemiologic, health economics, program efficiency, and policy outcome measures, in addition to programmatic feasibility [10]. The first level of the framework assesses the VMMC program using epidemiologic and health economic measures such as VMMC effectiveness, cost-effectiveness, incidence rate reduction, magnitude of impact, and total program cost. The second level of the framework assesses measures of program efficiency and policy outcomes, using expansion path curves [10, 16] and policy frontier plots [10]. The third level of the framework is related to program feasibility. Definitions of the outcome measures for the different levels of the conceptual framework can be found in Table 1.

**Table 1:** Definitions of outcome measures in the three-level conceptual framework.

Measure	Definition
Level 1	
*Effectiveness of VMMC*	Total number of VMMCs per HIV infection averted
*Cost-effectiveness of VMMC*	Cost per HIV infection averted
*Scale of reduction in the risk of HIV exposure*	HIV incidence rate reduction
*Magnitude of impact*	Total number of HIV infections averted over a given time period
*Total VMMC program cost*	Total cost of the VMMC program
Level 2	
*Expansion path curve*	Examines the incremental change in total VMMC program cost relative to the incremental change in total number of HIV infections averted
*Program efficiency policy frontier plot*	Delineates the different possible policy domains based on the theme of maximizing program efficiency (maximizing gain while minimizing pain, Gain/Pain index*)
*Total impact policy frontier plot*	Delineates the different possible policy domains based on the theme of maximizing the total impact of the VMMC program (number of HIV infections averted)
Level 3	
*Programmatic feasibility*	Feasibility based on on-the-ground country experiences

Voluntary medical male circumcision (VMMC) program scenarios are assessed based on epidemiological and health economics measures (Level 1), program efficiency and policy outcome measures (Level 2), and programmatic feasibility (Level 3).

* Gain/Pain index: the proportional reduction in the total number of infections averted (Gain) over the proportional reduction in the total VMMC program cost (Pain). These proportions are assessed relative to the baseline scenario of targeting males aged 15–49 years.

### Data sources

The model was parameterized using current empirical epidemiological and natural history data from sub-Saharan Africa [10]. The country-specific time series of HIV prevalence data was obtained from estimates by the Joint United Nations Programme on HIV/AIDS (UNAIDS) for 1990–2012 [17]. The baseline male circumcision rate of 10.3%, reflecting background non-VMMC program circumcisions, was obtained from Zimbabwe’s *Demographic and Health Survey* (DHS) 2005–06 [18], a nationally-representative household-based survey [19]. HIV prevalence data for each province in Zimbabwe were obtained from Zimbabwe’s DHS 2005–06 and DHS 2010–11 [3, 18]. Demographics were obtained from the database of the Population Division of the United Nations Department of Economic and Social Affairs [20]. The VMMC unit cost per age group used in the model was based on VMMC program data [10, 21]. We applied an annual discount rate of 3% to expenditures (VMMC cost) and savings (HIV infections averted) [22].

### VMMC program scenarios

#### Impact of Zimbabwe’s achieved VMMCs through the VMMC program

We estimated the impact of what has been achieved by the VMMC program to date by using the actual number of VMMCs that the program in Zimbabwe has implemented between 2009 and 2014 [9], but assuming no further VMMCs after 2014. The number of VMMCs in 2014 is based on the number of VMMCs done up to September 2014 [9]. We assessed the impact of the program using two assumptions for the age distribution of VMMCs. First, we assessed the impact using the current program data for the age distribution of VMMCs: that is, 71% of completed VMMCs are within the 15–49 age bracket and 29% are within the 10–14 age group [9]. Second, we assessed the VMMC program assuming that the circumcisions were delivered only within the target age bracket of 13–29 year old. In both scenarios we used a fixed VMMC rate, i.e. the likelihood of any male within a specific targeted subpopulation to be circumcised is uniform.

#### Baseline VMMC intervention scenario

In the modeled baseline intervention scenario (reference scenario), the scale-up of the VMMC program was initiated in 2010, with a “catch-up” phase up to 2017, and a “sustainability” phase up to 2025. The catch-up phase ends by reaching 80% VMMC coverage in the 15–49 age bracket. This coverage is maintained in the sustainability phase by circumcising the incoming cohorts into the 15–49 year old population. The scale-up of VMMC was assumed to be at a fixed rate.

This baseline VMMC scenario assumes 80% VMMC coverage among 15–49 year old males per the World Health Organization (WHO) and UNAIDS recommendation for VMMC scale-up plans [1, 2]. The scenario has been also adopted as the baseline of choice across countries in sub-Saharan Africa in the ongoing modeling efforts for assessing VMMC program efficiency gains through subpopulation prioritization [10, 23].

#### Prioritizing subpopulations

The scenarios for prioritizing specific subpopulations were based on age, geographic location, and the sexual risk profile of males. For each subpopulation scenario, 80% VMMC coverage by 2017 was assumed. Prioritization of a male subpopulation is meant here as intensifying demand creation and service availability for that specific subset of males. Prioritization is not meant to be an exclusion of VMMC services to anyone. These services need to be available to all males.

For the age-group prioritization scenarios, we prioritized each of the five-year age bands (10–14, 15–19… 45–49) and also wider age brackets (such as 10–29, 13–29, 15–24, and 15–29, among others). Two different VMMC scale-up scenarios were considered in prioritizing the 13–29 year old age bracket, which is Zimbabwe’s current age target. In the first scenario, the prioritization was informed by current program data for the age distribution of VMMCs in Zimbabwe—the distribution of VMMCs to males younger than 15 versus males older than 15 [9]. That is, we assumed that 29% of all VMMCs are within the age group 13–14 year old and the remaining 71% are among males 15–29 year old [9]. In the second scenario, the 13–29 year old age bracket was targeted using a fixed VMMC rate regardless of age within this age bracket.

For the geographic prioritization scenarios, the VMMC intervention was prioritized to 15–49 year old males based on the distribution of HIV infection across the provinces in Zimbabwe, with each province prioritized separately.

For the sexual risk-group prioritization scenarios, the VMMC intervention was prioritized to 15–49 year old males based on their sexual risk behavior profile—that is, according to their specific sexual risk group.

### Uncertainty analysis

We conducted a multivariate uncertainty analysis to specify the range of uncertainty in the effectiveness of VMMC with respect to variations in the structural parameters of the model. Monte Carlo sampling from uniform probability distributions was used for the uncertainty in the biological and behavioral parameters of the model. We assumed an uncertainty of 20% around the point estimates of all parameters. Each set of new parameters was used to refit HIV prevalence time series data in Zimbabwe. We implemented 500 uncertainty runs for each modeled intervention scenario to derive the mean value and associated 95% uncertainty interval for the effectiveness of the VMMC program.

## Results

### Impact of the current VMMC program scale-up in Zimbabwe

By September 2014, Zimbabwe circumcised 364,185 males. Assuming the current age distribution of VMMCs, the program is estimated to avert 40,301 HIV infections by 2025. If these VMMCs had been delivered exclusively within the official target age group of 13–29 year old, the program would instead avert an estimated 44,022 HIV infections by 2025, nearly 10% more infections.

In order to achieve 80% VMMC coverage by 2017 (the catch-up phase) among the 13–29 year old males, a total of 2.17 million VMMCs are required (Table 2). An additional 1.30 million VMMCs would also be required between 2018 and 2025 to maintain the 80% coverage. Accordingly, about 314,000 HIV infections would be averted between 2010 and 2025. The total cost of the VMMC program by 2025 would be US$293 million (all subsequent references to currency are in U.S. dollars). The number of VMMCs (by 2025) needed to avert one HIV infection (effectiveness) is 11, while the cost per infection averted (cost-effectiveness) is $934.

**Table 2:** Epidemic impact of prioritizing different age-group bands and brackets through the voluntary medical male circumcision (VMMC) program.

Age group	#VMMC/HIA (2010–25)	#VMMCs (2010–17) (millions)	Additional VMMCs (2018–25) (millions)	HIA (millions) (2010–25)	Cost (USD)/HIA (2010–25)	Total cost (billion) (2010–25)
15–49	11	2.52	1.1	0.33	1,010	0.33
13–29	11	2.17 (86%)	1.3	0.31 (96%)	934 (92%)	0.29 (89%)
13–29*	12	2.32 (92%)	1.3	0.29 (88%)	1,035 (102%)	0.30 (90%)
10–14	19	1.32 (52%)	1.3	0.14 (41%)	1,483 (147%)	0.20 (61%)
15–19	11	1.17 (46%)	1.2	0.20 (63%)	917.32 (91%)	0.19 (57%)
20–24	10	0.99 (39%)	1.0	0.21 (64%)	811 (80%)	0.17 (51%)
25–29	12	0.80 (32%)	0.9	0.14 (43%)	1,059 (105%)	0.15 (45%)
30–34	15	0.63 (25%)	0.7	0.09 (28%)	1,377 (136%)	0.12 (38%)
35–39	19	0.49 (20%)	0.6	0.06 (17%)	1,857 (184%)	0.10 (31%)
40–44	28	0.39 (15%)	0.5	0.03 (10%)	2,769 (247%)	0.09 (26%)
45–49	53	0.28 (11%)	0.4	0.01 (4%)	5,518 (546%)	0.07 (21%)
15–24	11	1.57 (62%)	1.3	0.27 (83%)	873 (86%)	0.23 (70%)
15–29	11	1.86 (74%)	1.3	0.30 (91%)	888 (88%)	0.26 (78%)
15–34	11	2.08 (83%)	1.2	0.31 (96%)	915 (91%)	0.39 (87%)
10–24	13	2.19 (87%)	1.5	0.28 (86%)	1,061 (105%)	0.29 (88%)
10–29	13	2.49 (99%)	1.5	0.31 (96%)	1,039 (103%)	0.32 (97%)
10–34	12	2.72 (108%)	1.4	0.33 (102%)	1,044 (103%)	0.35 (105%)
10–49	13	3.14 (125%)	1.3	0.35 (107%)	1,118 (111%)	0.44 (131%)

The number of VMMCs needed to avert one HIV infection (2010–2025) (*effectiveness*), the total number of VMMCs needed to reach 80% coverage by 2017, the additional number of VMMCs needed during the sustainability phase (2018–2025), the total number of HIV infections averted (2010–2025) (*magnitude of impact*), the cost needed to avert one HIV infection (2010–2025) (*cost-effectiveness*), and the total program cost (2010–2025) (*program cost*). Targeting the 15–49 year old male population is used as the baseline VMMC intervention scenario for comparison purposes. The numbers in parentheses indicate the fractions achieved relative to the baseline.

* 29% of all VMMCs are among males 13–14 year old and 71% are among males 15–29 year old.

VMMC: Voluntary medical male circumcision, HIA: HIV infection(s) averted.

Assuming the current age distribution of VMMCs [9], with 29% of VMMCs delivered to 13–14 year old and 71% to 15–29 year old, about 287,000 HIV infections would be averted between 2010 and 2025, the number of VMMCs needed to avert one infection is approximately 12, and the cost per infection averted is $1,035.

### Impact of the VMMC program in Zimbabwe with subpopulation prioritization to enhance program efficiency

#### Baseline VMMC intervention scenario

By targeting 15–49 year old males, 2.52 million VMMCs are required in the catch-up phase to achieve 80% VMMC coverage by 2017 (Table 2). Moreover, 1.12 million additional VMMCs would be required between 2018 and 2025 to maintain the 80% coverage. Approximately 326,000 HIV infections would be averted between 2010 and 2025. The total cost of the VMMC program by 2025 would be $326 million. The number of VMMCs (by 2025) needed to avert one HIV infection is approximately 11, while the cost per infection averted is $1,010.

#### Prioritization by age-group: Epidemiologic and health economic measures

**Fig 1:**
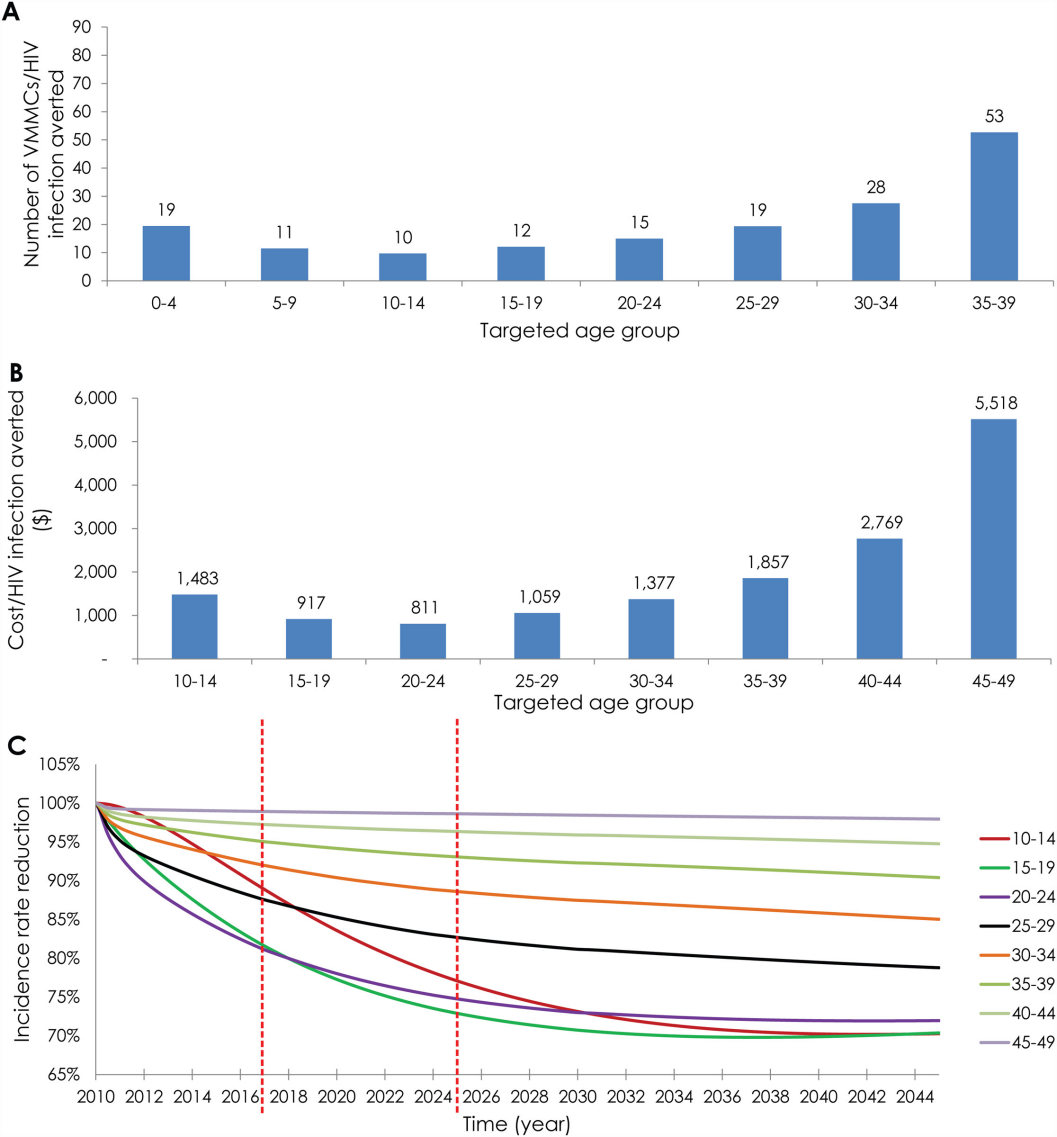
Projected outcomes of age-group prioritization. **A)** Number of voluntary medical male circumcisions (VMMCs) needed to avert one HIV infection (*effectiveness*) by 2025. **B)** Cost per HIV infection averted by 2025 (*cost-effectiveness*). **C)** Projected incidence rate reduction throughout the years up to 2045. The results are for 80% VMMC coverage by 2017 in each of the prioritized age band.

Fig 1 illustrates the projected outcomes of age-group prioritization. When we targeted each of the five-year age bands separately, in the intermediate term—by 2025—the number of VMMCs needed to avert one HIV infection ranged from 10 to 53 (Fig 1A). Prioritizing males in the 20–24 age group achieved the highest effectiveness: 10 VMMCs were needed per HIV infection averted, which is better than the effectiveness achieved by targeting 13–29 year old males (11 VMMCs per infection averted). Prioritizing males in the 45–49 age group was least effective: 53 VMMCs were needed per infection averted (Fig 1A). In the long term—by 2045—the optimal effectiveness was still achieved by prioritizing the 20–24 age group (10 VMMCs per infection averted), while the lowest effectiveness was achieved by prioritizing males in the 45–49 age group (67 VMMCs per infection averted).

Fig 1B illustrates the age-stratified cost-effectiveness. In the intermediate term, the VMMC program cost per infection averted ranged from $811 (20–24 age group) to $5,518 (45–49 age group). In the long term, the VMMC program cost per infection averted ranged from $633 (20–24 age group) to $5,019 (45–49 age group).

Fig 1C illustrates the impact of age-group prioritization on HIV incidence rate in the adult population (15–49 year old) throughout the years up to 2045. By 2017, the largest reductions in HIV incidence rate (about 19%) were observed by targeting males in the 15–19 and/or 20–24 age groups. By 2025, the largest reductions in incidence rate (about 27%) were observed by targeting males 10–14, 15–19, and/or 20–24 year old. By 2045, as much as a 30% reduction was observed by also targeting males 10–14, 15–19, and/or 20–24 year old.

#### Prioritization by age-group: Program efficiency and policy outcome measures

**Fig 2:**
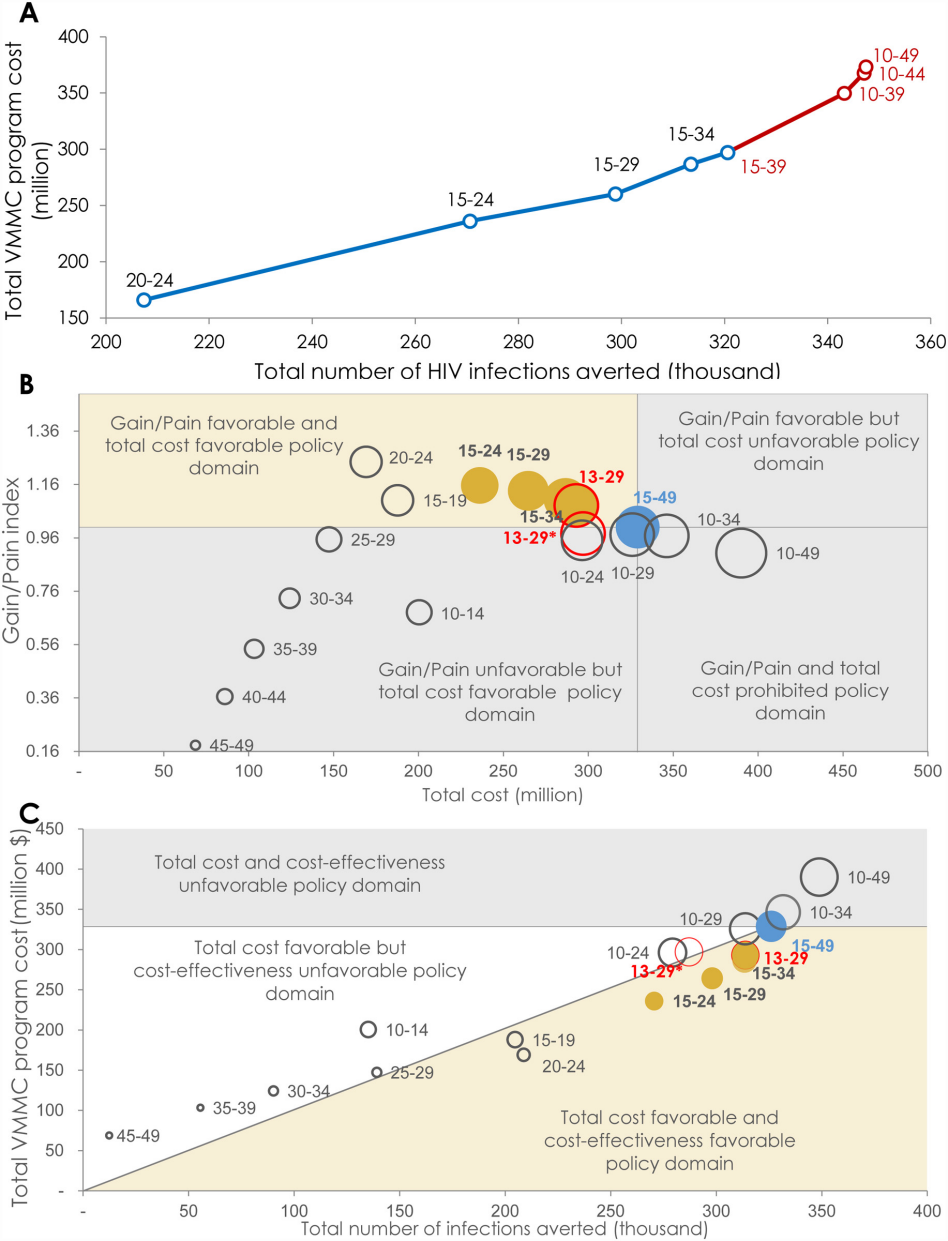
Program efficiency and policy domains of age-group prioritization in the voluntary medical male circumcision (VMMC) program. **A)** Expansion path curve showing the incremental change in total cost of the VMMC program (*program cost*) relative to the incremental change in total number of HIV infections averted (*magnitude of impact*) for each age group- targeted scenario. The blue line shows the expansion of the program with minimal diminishing of returns, and the red line shows the expansion of the program with considerable diminishing of returns. **B)** Frontier policy plot delineating the different policy domains based on the theme of maximizing program efficiency (maximizing gain while minimizing cost). Circle size represents the total number of HIV infections averted (*magnitude of impact*). **C)** Frontier policy plot delineating the different policy domains based on the theme of maximizing the total impact of the VMMC program. Circle size represents the total number of VMMCs needed. In both **B** and **C**, the orange circles represent the age brackets that fit into the optimal policy domain, the red circles represent Zimbabwe’s current targeted age group (13–29 year old males), and the blue circle represents the baseline VMMC intervention scenario. * Gain/Pain index: the proportional reduction in the total number of infections averted (Gain) over the proportional reduction in the total VMMC program cost (Pain). These proportions are assessed relative to the baseline scenario of targeting males aged 15–49 years.

Fig 2A shows the expansion path curve for prioritizing by age-group. The expansion path starts by targeting the 20–24 year old population—the age group that has the highest effectiveness (Fig 1A). The other five-year age groups are then added based on a hierarchy of decreasing effectiveness. Though the number of infections averted increased with each age-group expansion, the total cost also increased and at a higher rate (Fig 2A). As the expansion reached males older than 35 years of age or younger than 15 years of age (10–14 year old), the diminishing returns of the expansion became evident, highlighting the decline in VMMC program efficiency.

Fig 2B provides an assessment of program efficiency—by 2025—through the “Gain/Pain index”. The “Gain/Pain index” is defined as the proportional reduction in the total number of infections averted (Gain) over the proportional reduction in the total cost of the VMMC program (Pain) [10]. These proportions were assessed relative to the baseline scenario (targeting the 15–49 year old males). By targeting males 15–19, 20–24, 15–24, 15–29, and 15–34 year old, the Gain/Pain index was >1, affirming that this age range contains the subpopulations with the highest program efficiency. This measure was also >1 when the 13–29 age bracket was targeted using a fixed VMMC rate. When the targeting of 13–29 year old was done using the current age distribution of VMMCs, as per program data [9], the Gain/Pain index became slightly <1. This measure was also <1 by targeting males 10–14, 25–29, 30–34, 35–39, 40–44, 45–49, 10–24, 10–29, 10–34, and 10–49 year old.

Fig 2B also shows the policy-favorable domain using a policy frontier plot. The policy theme here is maximizing program efficiency, thereby showing the Gain/Pain index at different scales of the VMMC program. The optimal policy domain is the domain of favorable program efficiency (Gain/Pain index > 1) and also favorable total program cost (total program cost being less than that of the baseline intervention scenario). The optimal policy domain was obtained by prioritizing 15–19, 20–24, 15–24, 15–29, and/or 15–34 year old males. Targeting the 13–29 age bracket using a fixed VMMC rate also fitted into the optimal policy domain. However, program efficiency of this age bracket became slightly unfavorable when the current age distribution of VMMCs (as per program data) was taken into account. The program’s efficiency also became unfavorable when 10–14 year old males were added to the prioritized age groups in the 10–24 and 10–29 age brackets. The program’s total cost became unfavorable (total program cost being equal to or higher than that of the baseline intervention scenario) when the 10–34 and 10–49 year old cohorts were targeted. Other age-group targeting schemes were unfavorable for program efficiency and/or total program cost (Fig 2B).

Fig 2C explores different policy domains by examining the total impact, total program cost, and cost-effectiveness domains by 2025. The policy theme here is maximizing the total impact of the VMMC program. The optimal policy domain is the domain of favorable total program cost (less than that of the baseline intervention scenario), favorable cost-effectiveness (more cost-effective than that of the baseline intervention scenario), and most importantly, favorable magnitude of impact (total number of infections averted nearly as large as that of the baseline intervention scenario). The optimal policy domain was obtained by prioritizing males 15–19, 20–24, 15–24, 15–29, and/or 15–34 year old. Targeting the 13–29 age bracket using a fixed VMMC rate also fitted into the optimal policy domain. Targeting the 13–29 age bracket using the current age distribution of VMMCs did not undermine the favorability of the magnitude of impact nor total program cost, but the cost-effectiveness was unfavorable—it was slightly lower than that of the baseline scenario. Adding 10–14 year old males to these targeted age groups, as well as the remaining age-group targeting schemes, rendered the VMMC program unfavorable for magnitude of impact, total program cost, and/or cost-effectiveness (Fig 2C).

Table 2 summarizes the quantitative results of the different targeting schemes used in generating the program efficiency and policy domain figures for the age-group prioritization (Fig 2B and 2C). By targeting the 20–24 age group, 64% of the magnitude of the impact of the baseline scenario (number of infections averted by 2025) was achieved with 61% fewer VMMCs (by 2017) and 49% lower total program cost (by 2025). By targeting the 15–24 age bracket, 83% of the magnitude of the impact of the baseline scenario was achieved (by 2025) with 38% fewer VMMCs (by 2017) and 30% lower total program cost (by 2025). By targeting the 13–29 age bracket with a fixed VMMC rate, 96% of the magnitude of the impact of the baseline scenario was achieved (by 2025) with 14% fewer VMMCs (by 2017) and 11% lower total program cost (by 2025).

#### Prioritization by geographic location

**Fig 3:**
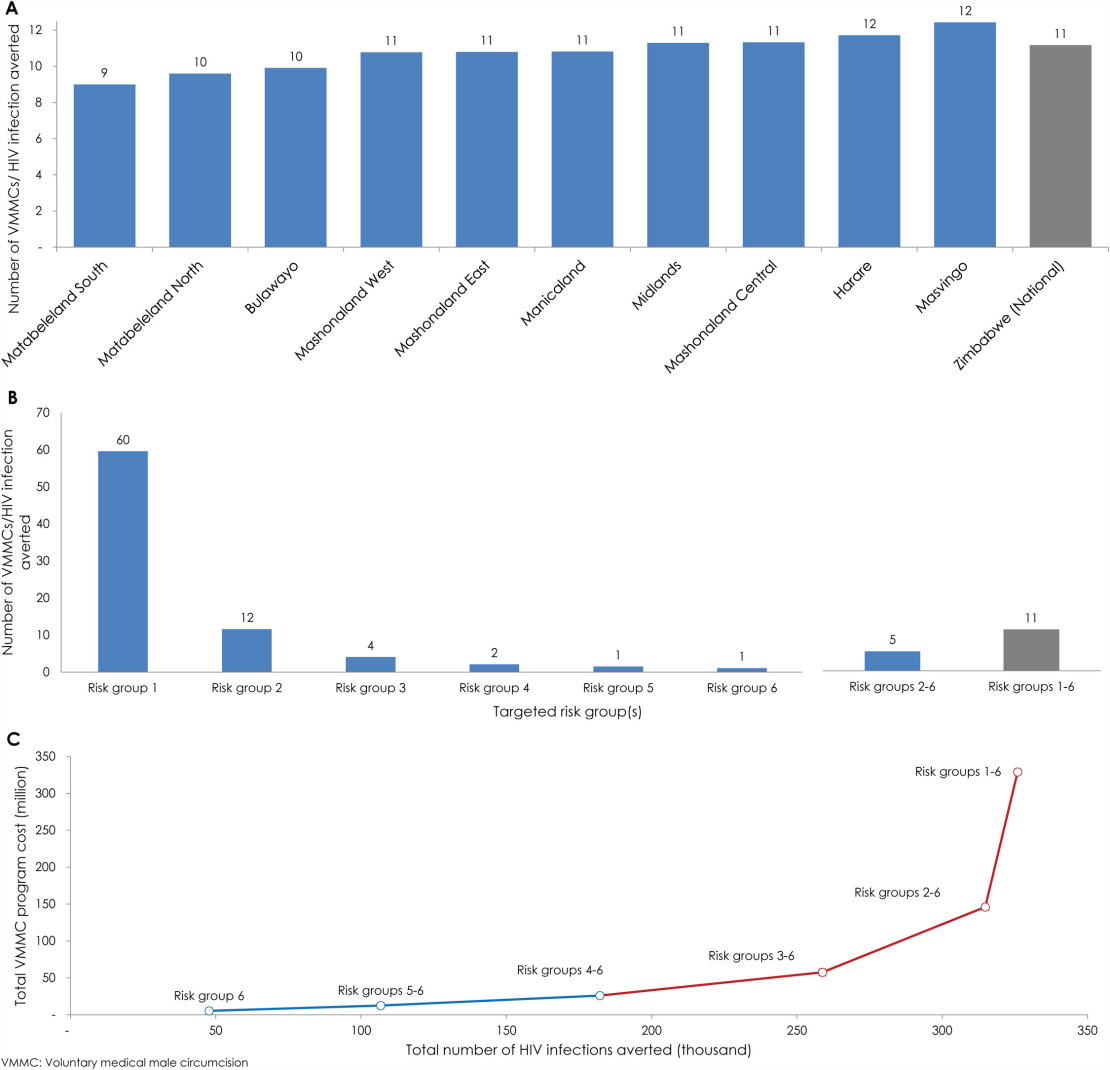
Projected outcomes of geographic and sexual risk-group prioritization. **A)** Number of voluntary medical male circumcisions (VMMCs) needed to avert one HIV infection (*effectiveness*) by 2025 through geographic prioritization. **B)** Number of VMMCs needed to avert one HIV infection by 2025 through risk-group prioritization. **C)** Expansion path curve showing the incremental change in total cost of the VMMC program relative to the incremental change in total number of HIV infections averted for each sexual risk-group targeted scenario. The blue line shows the expansion of the program with minimal diminishing of returns, and the red line shows the expansion of the program with considerable diminishing of returns.

Fig 3A shows the effectiveness of geographic prioritization. In the intermediate term—by 2025—the effectiveness of this strategy ranged from nine to 12 VMMCs per HIV infection averted. The highest effectiveness was achieved by targeting Matabeleland South, Matabeleland North, and Bulawayo—the provinces with the highest HIV prevalence in Zimbabwe. The lowest effectiveness was achieved by targeting Masvingo and Harare. The variation in VMMC effectiveness across the provinces was minor, producing a simplistic linear expansion path curve (not shown).

#### Prioritization by sexual risk-group

Fig 3B and 3C show the projected outcomes of sexual risk-group prioritization by 2025. In comparison to the other subpopulation prioritization schemes, the effectiveness of risk-group prioritization varied immensely by group (Fig 3B). In the baseline scenario, where all risk groups were targeted, 11VMMCs were required to avert one HIV infection. Meanwhile, the effectiveness of targeting by specific risk group ranged from one to 60 VMMCs per infection averted. The highest effectiveness was achieved by targeting males in the highest risk group (risk group 6): only one VMMC was needed to avert one infection. In contrast, targeting males in the lowest risk group (risk group 1) required 60 times more VMMCs per infection averted. By targeting risk groups 2–6 (that is, excluding only the lowest sexual risk group), the effectiveness was 5 VMMCs per infection averted, in comparison to 11 VMMCs per infection averted by targeting all risk groups together.

Fig 3C shows the expansion path curve for prioritizing by risk group. The incremental change in total cost of the VMMC program relative to the incremental change in total number of HIV infections averted was highly nonlinear with the addition of each risk group. Returns diminished rapidly with the expansion of the VMMC program to males whose sexual behavior puts them at lower risk of acquiring HIV.

### Uncertainty analysis

**Fig 4:**
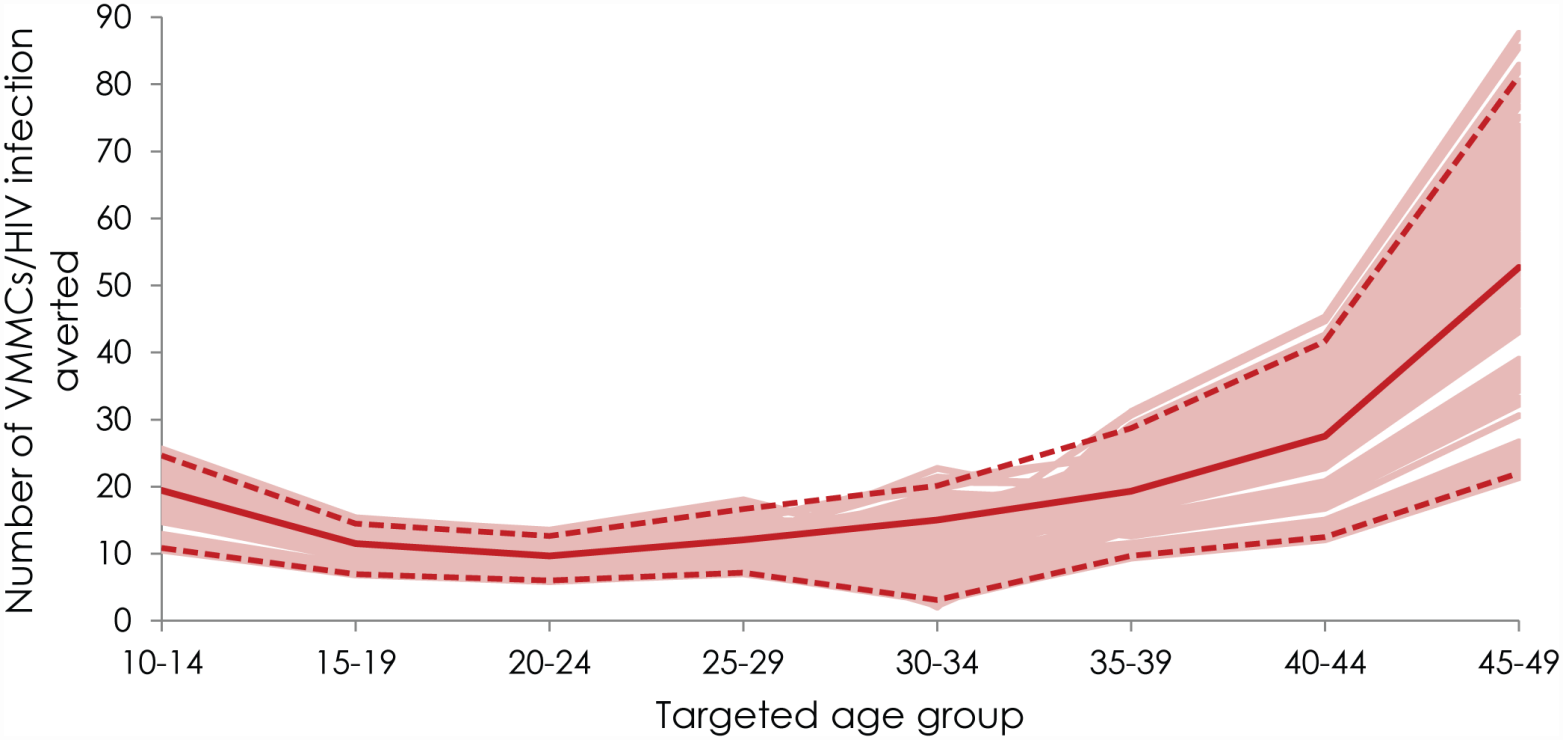
Range of uncertainty for the number of voluntary medical male circumcisions (VMMCs) needed to avert one HIV infection by 2025 for the different prioritized age groups. The solid red line represents the point estimate curve. The dashed lines bracket the 95% uncertainty interval of the curves generated in the uncertainty analyses.

Fig 4 shows the range of uncertainty for the number of VMMCs needed to avert one HIV infection by 2025 for the different prioritized age groups. The figure shows the curves from all uncertainty runs along with the point estimate curve and 95% uncertainty interval. Overall, the curves followed the same U-shaped pattern as the point estimate curve for the effectiveness of age-group prioritization. For the majority of uncertainty runs, the highest effectiveness was achieved by prioritizing 20–24 year old males.

## Discussion

Our findings demonstrate that the current VMMC program in Zimbabwe targets an efficient and impactful age bracket (13–29 year old) and is averting a significant number of HIV infections. However, the epidemiological and health economic benefits of the program can be maximized by intensifying demand creation and service availability for specific subpopulations, while maintaining VMMC service availability to all adult males. Program efficiency and public health and economic benefits of VMMC scale-up can be improved by giving priority to young sexually active males and males whose sexual behavior puts them at higher risk of acquiring HIV.

Since the start of the VMMC initiative in 2009, VMMC scale-up in Zimbabwe has witnessed an accelerated uptake among males. However, nearly 29% of the completed VMMCs to date are within the 10–14 age group [9]—a group with less programmatic efficiency. With only the completed VMMCs, and their current age distribution, the program is expected to avert 40,301 HIV infections by 2025. This is 10% fewer infections averted (40,301 compared to 44,022) than would have been averted if the program had delivered these circumcisions only within the target age bracket of 13–29 year old males. Our findings from this reevaluation of the program have important implications, given the rising programmatic feasibility challenges and funding constraints [23]. Focusing the program on the most impactful circumcisions through subpopulation prioritization is a key strategy for achieving greater program efficiency.

Based on the different outcome measures within the three-level conceptual framework (Table 1), the optimal public health benefits of VMMC are dependent on the age at which males undergo circumcision. In comparison with the baseline scenario (targeting males who are 15–49 year old), prioritizing males in the 20–24 age group enhances the program’s effectiveness by up to 13% (by 2025; Fig 1A), and its cost-effectiveness by up to 20% (Fig 1B). In comparison with the results generated for Zimbabwe’s current age target (13–29 year old) with the actual current VMMC distribution by age, giving priority to males in the 20–24 age group enhances VMMC effectiveness by 23% and cost-effectiveness by 22%.

If the program aims to achieve the largest reduction in HIV incidence rate in the short term (by 2017), the optimal age groups to prioritize are 15–19 and 20–24 year old. If the program aims to achieve the largest reduction in incidence rate in the intermediate or long term (by 2025 or 2045), the optimal age groups to prioritize are 10–14, 15–19, and 20–24 year old. Thus, by reaching males during or immediately prior to the age at which HIV incidence rate peaks, the VMMC program in Zimbabwe can optimize the public health benefits of VMMC.

As part of reevaluating the VMMC program plan, the expansion path curve delineated how Zimbabwe’s VMMC program can be expanded efficiently to include more age groups, as feasible by total VMMC program cost (Fig 2A). With expansion, the diminishing returns became evident as males older than 35 years of age or younger than 15 years of age were included. The policy frontier plots, with both the program efficiency theme and the total impact theme, have also converged on the same optimal age groups: 15–24, 15–29, or 15–34 (Fig 2B and 2C). The policy frontier plots also indicated that the 13–29 age bracket is programmatically efficient, though slightly inferior to the optimal age brackets.

The VMMC program in Zimbabwe could also focus on improving program efficiency through geographic prioritization. The efficiency of the program can be enhanced by prioritizing geographic areas (e.g. provinces) whose HIV prevalence exceeds that of the national HIV prevalence. VMMC effectiveness can be improved by as much as 19% (by 2025) by giving priority to Matabeleland South, Matabeleland North, and Bulawayo—the provinces with the highest HIV prevalence (Fig 3A). However, since the variation in HIV prevalence across the provinces is rather small [3, 18], prioritizing by province did not lead to major gains in VMMC program efficiency.

Despite the challenges in identifying males whose sexual behavior puts them at higher risk of acquiring HIV [10], substantial gains in program efficiency can be realized by focusing on this population. VMMC effectiveness can be increased by an order of magnitude by prioritizing males with the highest sexual-risk behavior (by 2025; Fig 3B). Even simple targeting by risk behavior can highly increase program efficiency. Theoretically, by excluding males in the group at lowest risk, 97% of the impact of the baseline intervention plan (by 2025) could be achieved with 55% fewer VMMCs (by 2017) and 54% lower total cost (by 2025). The intervention among the highest risk group is highly effective because of a direct effect, VMMC efficacy in preventing acquisition, as well as an indirect effect, preventing the onward chains of transmission from the averted high-risk infections. Identifying, without stigmatizing, people at higher risk of HIV infection remains a major challenge in HIV prevention. However, a potential consideration for the VMMC program is to develop approaches by which the program can reach clients of sex workers, by enlisting the sex workers themselves to serve as interpersonal communication agents. Moreover, a recent study from Rakai, Uganda, has demonstrated the feasibility of gender-specific and well-calibrated indices to predict the risk of HIV acquisition [24]. Such approaches may offer opportunities for substantial gains through prioritization by risk.

In a previous study, the ASM model and the three-level conceptual framework were applied to Zambia to investigate program efficiency gains through subpopulation prioritization [10]. Overall, by 2025 VMMC effectiveness in Zimbabwe is slightly higher than that in Zambia (11 versus 12 VMMCs per HIV infection averted). This is because HIV prevalence in Zimbabwe is higher over the predicted time course of the epidemic than it is in Zambia. However, with the similarity in the age distribution of HIV incidence rate, the optimal age groups to be prioritized in Zambia and Zimbabwe are identical. Targeting by risk group also manifested major gains in program efficiency in both countries, but the programmatic feasibility of this approach remains to be explored and determined. The outcomes of geographic prioritization by province were different in the two countries. By 2025, this strategy improved VMMC effectiveness by as much as 33% in Zambia versus 19% in Zimbabwe. That is because the HIV epidemic in Zambia is more heterogeneous across the geography of this country than it is in Zimbabwe.

A key consideration influencing our model projections is the uncertainty surrounding future HIV incidence projections. It is not possible to precisely quantify the scale of uncertainty in HIV incidence estimates. Different scale-up plans for ART and other HIV interventions can change HIV incidence projections, thereby potentially affecting the outcome of Zimbabwe’s VMMC program. Nevertheless, in our study for Zambia [10], we showed that even within the context of an optimistic ART scale-up scenario [25], VMMC will remain an impactful intervention. The analysis for Zambia has shown that twice as many VMMCs would be needed to avert one HIV infection in presence of ART scale-up [10]. However, this did not undermine the fact that VMMC will remain a cost-effective intervention for HIV hyper-endemic settings such as Zambia and Zimbabwe. The results of subpopulation prioritization were also unaffected by ART scale-up.

We used an elaborate mathematical model to capture the complexity of HIV dynamics, but modeling predictions can depend on model structure and the accuracy of the data used to parameterize the model. Our study did not cover all aspects of VMMC scale up, such as those related to logistical feasibility, social dimension, and community relationships. The modeling results presented here are conditioned on the representativeness of the data input, such as time-trend data for HIV prevalence, baseline male circumcision prevalence, demographics, VMMC unit costs, and discount rates. To address the uncertainty in model input, we conducted a multivariate uncertainty analysis to assess the impact of changes in the structural and biological parameters of the model (Fig 4). This analysis supported the validity of our results (Fig 4).

## Conclusions

The ASM modeling tool and the three-level conceptual framework implemented here provide an opportunity for more strategic programming in Zimbabwe by focusing on the efficient use of limited resources. This approach was implemented to demonstrate the optimal public health and economic benefits of making subpopulations the focus of VMMC services and demand creation—results that can inform national policy and programming. Zimbabwe may decide to intensify demand creation and service availability for specific subpopulations to optimize VMMC’s epidemiological and health economics impact. The current VMMC program plan in Zimbabwe is already targeting an efficient and impactful age bracket (13–29 year old), but not the optimal one. Prioritizing males between the ages 15–34 years will improve VMMC program efficiency. Efficiency can also be enhanced by prioritizing geographic areas with higher HIV prevalence than the national HIV prevalence, but the gains are not substantial. The program’s efficiency can be enhanced immensely by prioritizing males whose sexual behavior puts them at higher risk for acquiring HIV. Last but not least, any policy deliberations of the reevaluated targets should take into consideration programmatic feasibility on the ground.
